# Supplementary figures and images for: PLB-985 Neutrophil-Like Cells as a Model To Study Aspergillus fumigatus Pathogenesis
Source: mSphere. 2022 Jan 5;7(1):e00940-21. doi: 10.1128/msphere.00940-21 (PMC8730815; doi:10.1128/msphere.00940-21)

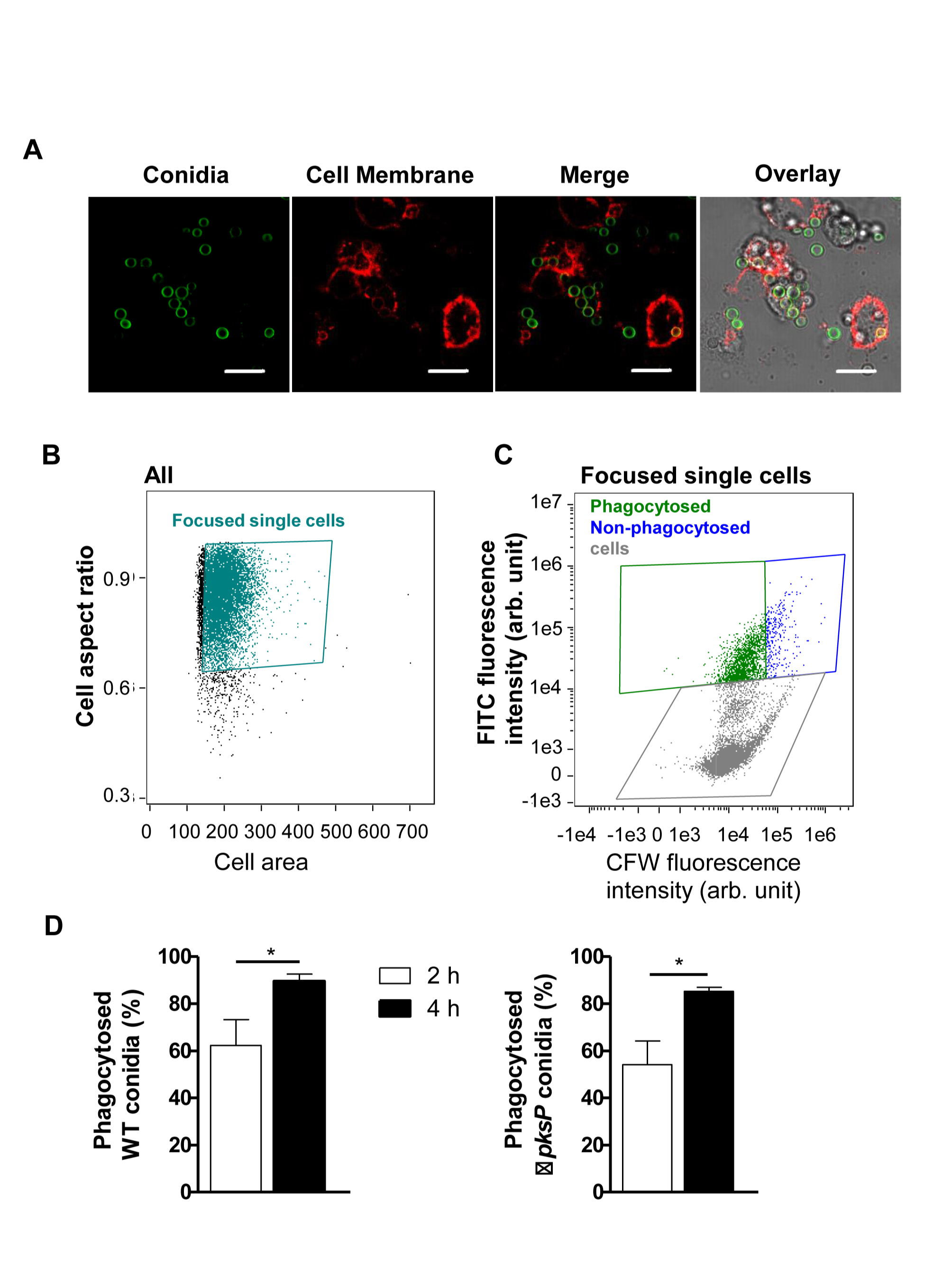

Supplement: FIG S1 [file msphere.00940-21-sf001.tif]

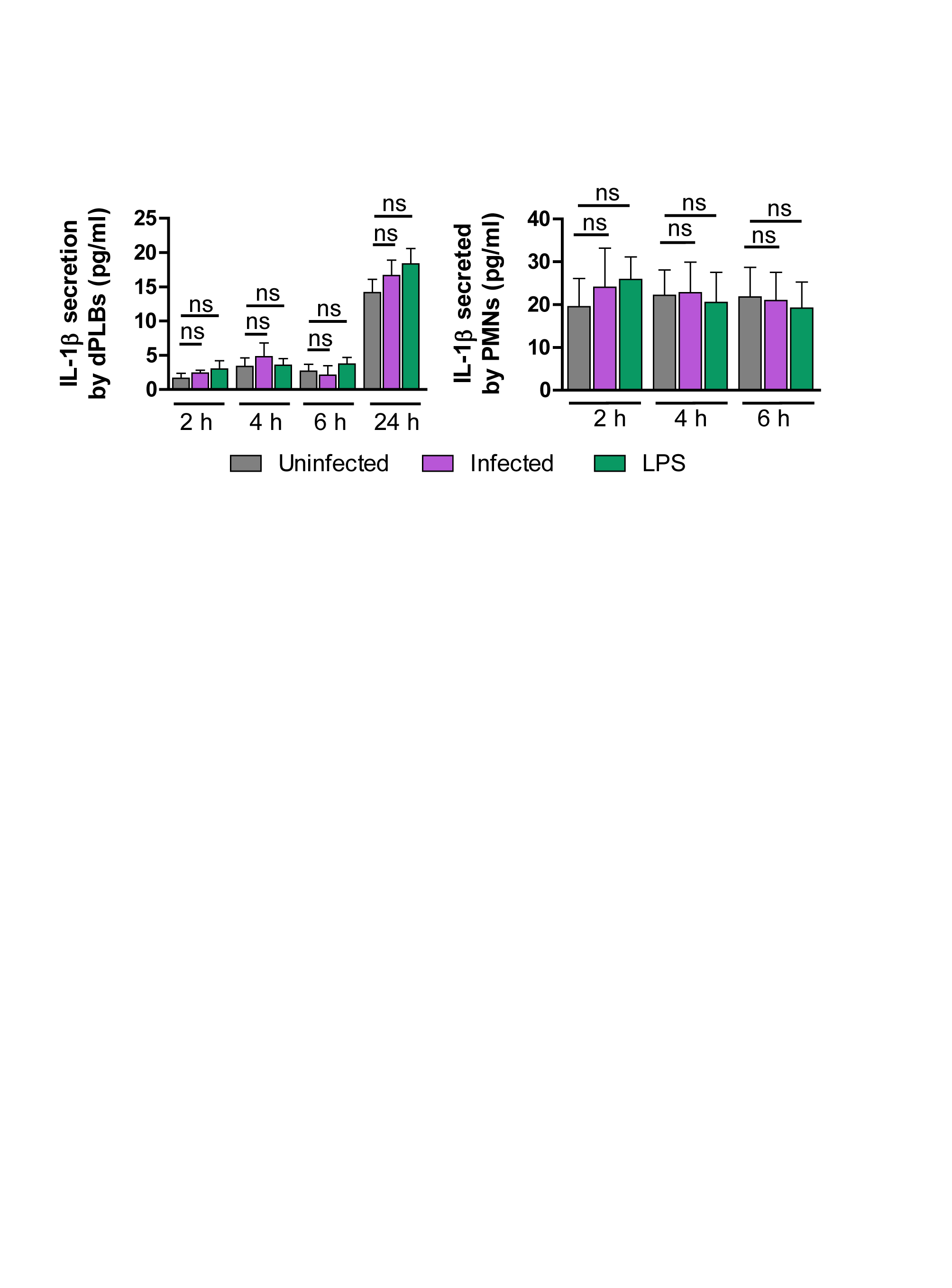

Supplement: FIG S2 [file msphere.00940-21-sf002.tif]

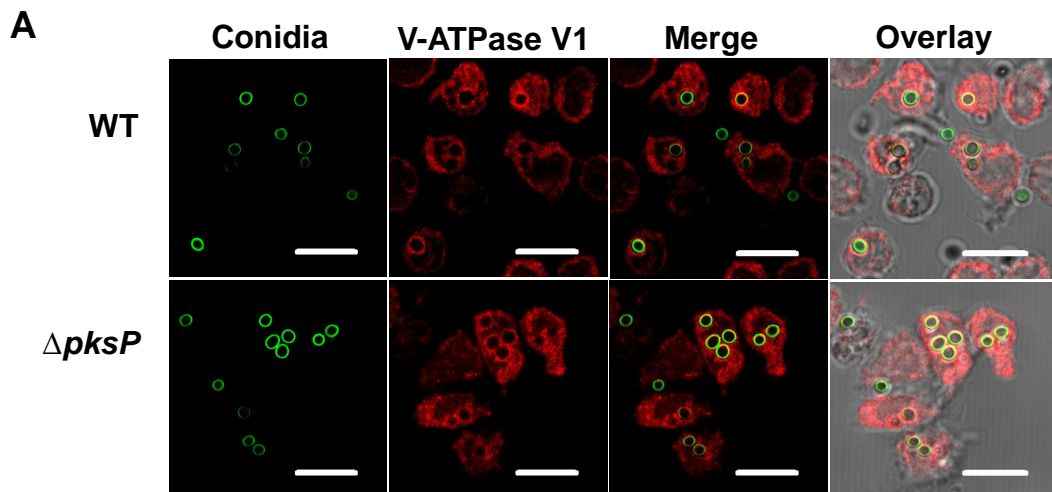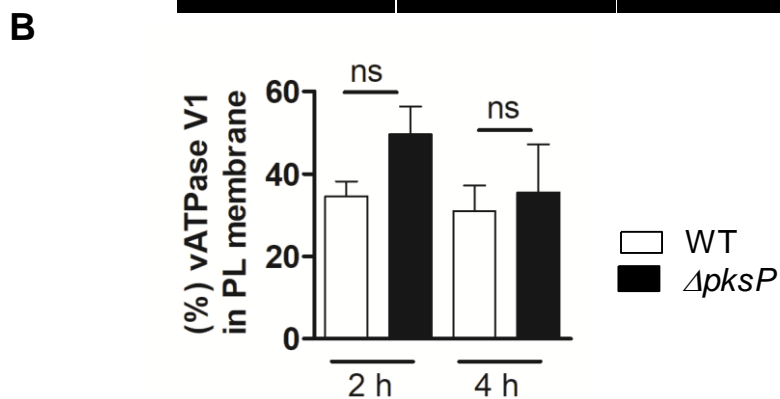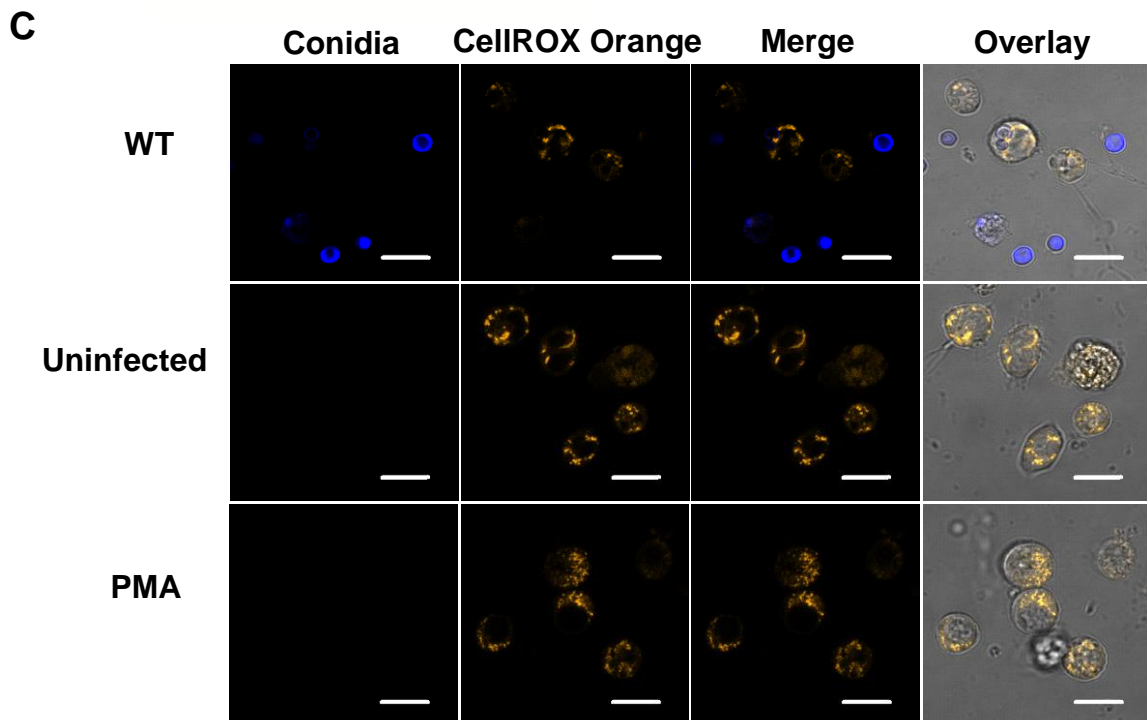

Supplement: FIG S3 [file msphere.00940-21-sf003.pdf]

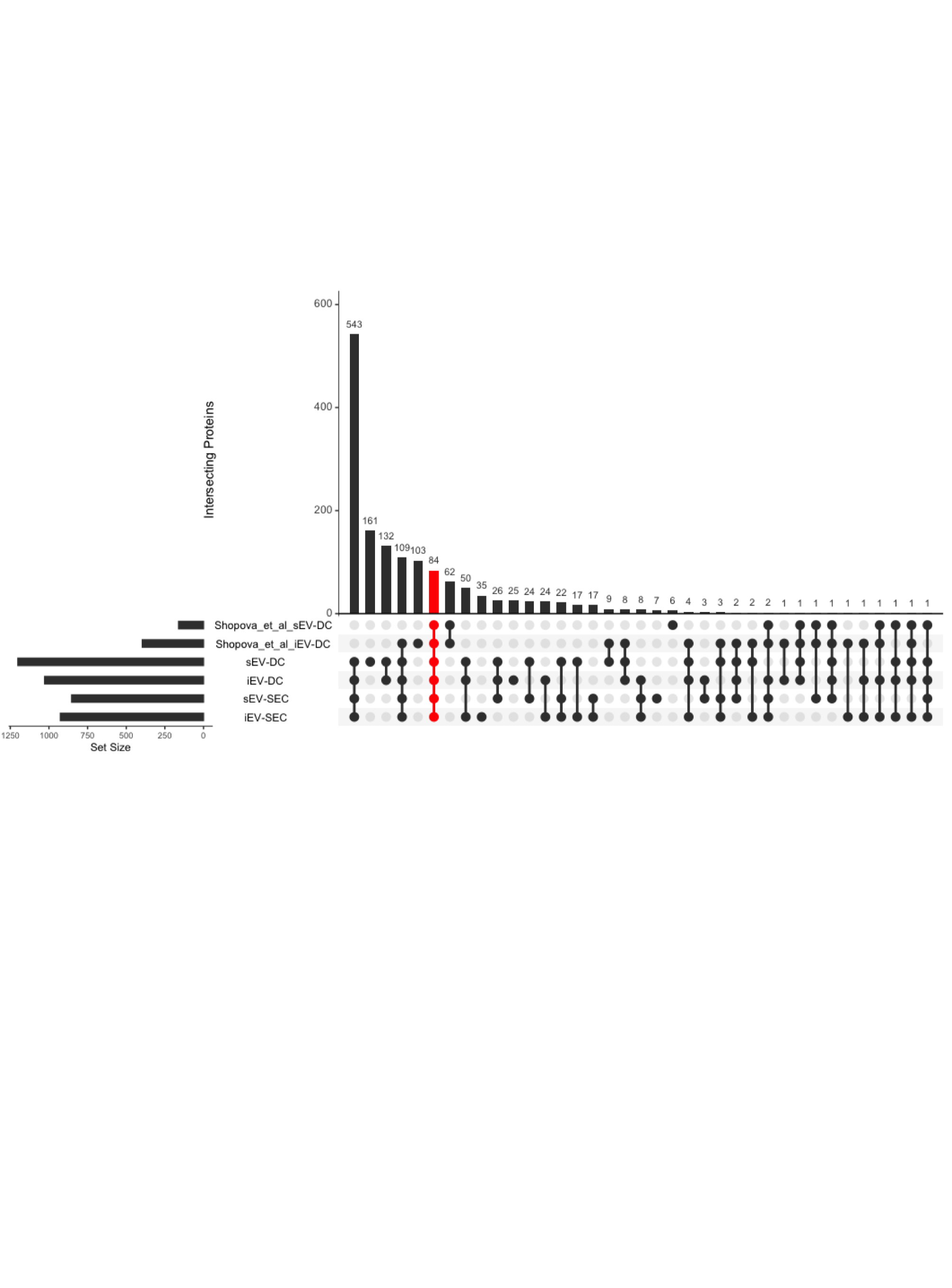

Supplement: FIG S4 [file msphere.00940-21-sf004.tif]

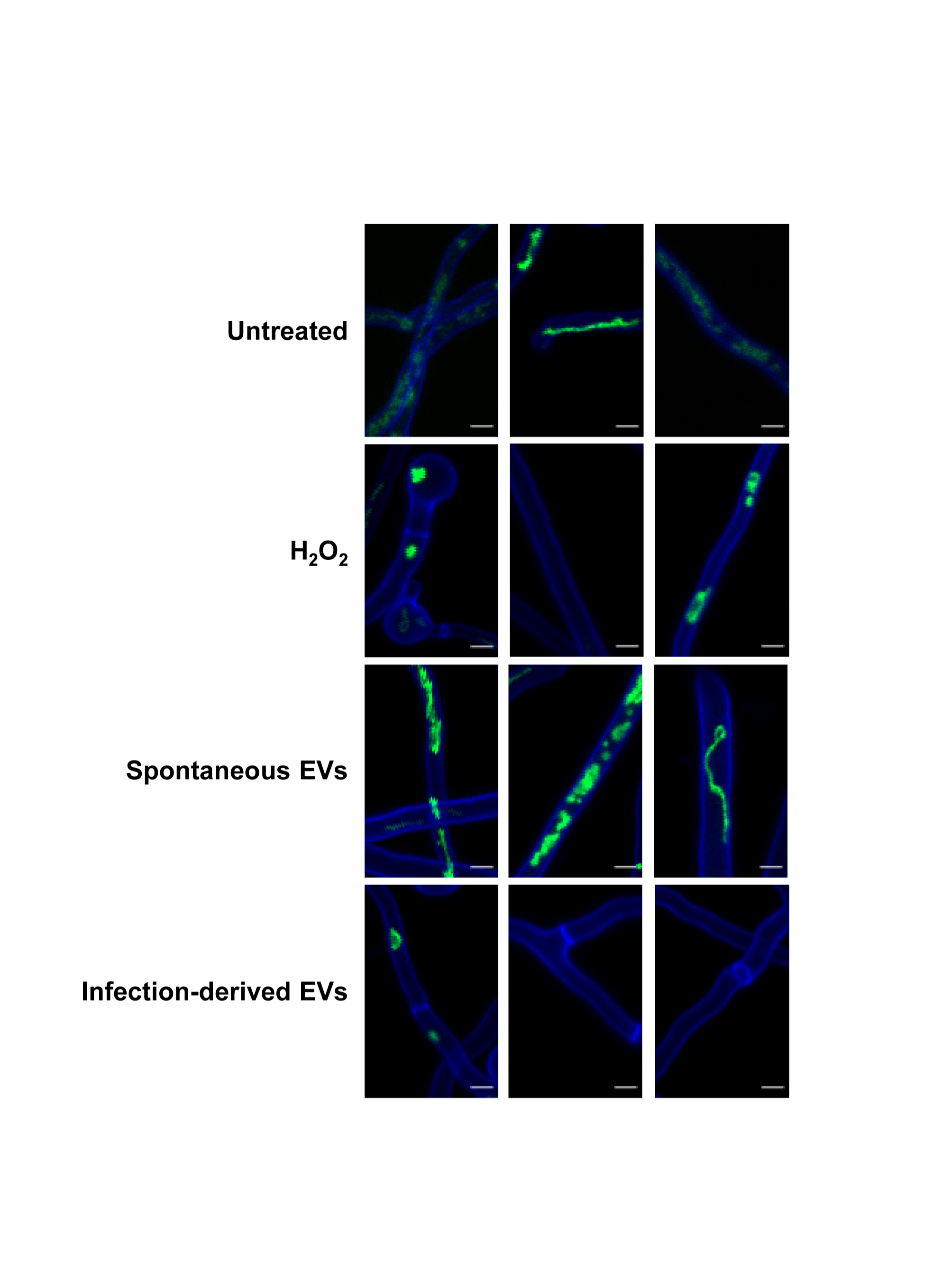

Supplement: FIG S5 [file msphere.00940-21-sf005.tif]
