# Supplementary material for: PLB-985 Neutrophil-Like Cells as a Model To Study Aspergillus fumigatus Pathogenesis
Source: mSphere. 2022 Jan 5;7(1):e00940-21. doi: 10.1128/msphere.00940-21 (PMC8730815; doi:10.1128/msphere.00940-21)
Supplement: TABLE S1 [file msphere.00940-21-st001.docx]

| **Gene** | **Accession** | **Description** | **(PSMs/AAs)*(Cov%/100)** |
| --- | --- | --- | --- |
| IGKC | P01834 | Immunoglobulin kappa constant | 3.45421 |
| APOA1 | P02647 | Apolipoprotein A-I | 1.73397 |
| IGHM | P01871 | Immunoglobulin heavy constant mu | 1.26349 |
| IGLC2 | P0DOY2 | Immunoglobulin lambda constant 2 | 0.82453 |
| IGHG1 | P01857 | Immunoglobulin heavy constant gamma 1 | 0.51688 |
| HBB | P68871 | Hemoglobin subunit beta | 0.43599 |
| IGHG2 | A0A286YEY4 | Immunoglobulin heavy constant gamma 2 (Fragment) | 0.27205 |
| APOB | P04114 | Apolipoprotein B-100 | 0.26471 |
| IGHA1 | P01876 | Immunoglobulin heavy constant alpha 1 | 0.16677 |
| C1QB | P02746 | Complement C1q subcomponent subunit B | 0.16126 |
| NPTN | Q9Y639 | Neuroplastin | 0.12879 |
| APCS | P02743 | Serum amyloid P-component | 0.12233 |
| C1QC | P02747 | Complement C1q subcomponent subunit C | 0.07286 |
| APOH | P02749 | Beta-2-glycoprotein 1 | 0.06858 |
| APOD | P05090 | Apolipoprotein D | 0.06349 |
| EMB | D6RDX7 | Embigin | 0.04667 |
| CFH | P08603 | Complement factor H | 0.02729 |
| IGKV2-40 | A0A087WW87 | Immunoglobulin kappa variable 2-40 | 0.02545 |
| DNAJA1 | P31689 | DnaJ homolog subfamily A member 1 | 0.01884 |
| HPX | P02790 | Hemopexin | 0.01541 |
| C4B | F5GXS0 | C4a anaphylatoxin | 0.00783 |
| FN1 | P02751 | Fibronectin | 0.00560 |
| C3 | P01024 | Complement C3 | 0.00394 |
| C8A | P07357 | Complement component C8 alpha chain | 0.00394 |
| AHSG | P02765 | Alpha-2-HS-glycoprotein | 0.00229 |
| C1R | B4DPQ0 | Complement subcomponent C1r | 0.00118 |
